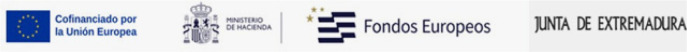# Technical-tactical performance of Spanish female basketball players in first division (2013–2022): effects of match outcome, location, and playing position

**DOI:** 10.5114/biolsport.2026.152349

**Published:** 2025-08-29

**Authors:** Carlos D. Gómez-Carmona, Elena Gómez-Ramos, María I. Piñar, José M. Contreras, Sergio J. Ibáñez

**Affiliations:** 1 Research Group in Training, Physical Activity and Sports Performance (ENFYRED), Faculty of Human and Social Sciences, University of Zaragoza, 44003 Teruel, Spain; 2 Research Group in Optimization of Training and Sports Performance (GOERD), Department of Didactics of Music, Plastic and Body Expression, Faculty of Sport Science, University of Extremadura, 10003 Caceres, Spain; 3 BioVetMed & SportSci Research Group. University of Murcia, 30100, Murcia, Spain; 4 Faculty of Sport Science, University of Granada, 18011 Granada, Spain; 5 Faculty of Education Sciences, University of Granada, 18071 Granada, Spain

**Keywords:** Contextual factors, Performance analysis, Game-related statistics, Elite level, Decision-making

## Abstract

Basketball performance is influenced by technical-tactical factors, but their effects across different contexts are not well understood in women’s basketball. This study analyzed the influence of match outcome (win, lose), match location (home, away), and playing positions (guard, G; point-guard, PG; small-forward, SF; power-forward, PF; center, C) on Key Performance Indicators (KPIs) in Spanish female basketball players. Data from 1786 games involving 897 players (33243 cases) across 10 consecutive seasons were analyzed (2013–2022). Sixteen normalized KPIs (standardized by minutes played) were evaluated using linear mixed modeling with individual player ID as a random factor, controlling for nested data structure (*ICC* > 0.10, p < 0.001). Fixed effects included playing position, match outcome, match location, and their interactions. Results revealed position-specific patterns: centers and power-forwards achieved significantly higher values in 2-point shots, offensive rebounds (C > PF > SF > PG=G), and blocks (C > PF > SF=PG=G), while guards obtained higher 3-point shooting (G=PG > SF=PF=C), assists (G > PG > SF=PF=C), and steals (G=PG > SF=PF=C) with large effect sizes. Winning teams significantly outperformed losing teams, with the largest differences in points, assists, and reduced turnovers. Home teams demonstrated significant advantages in assists, blocks, and reduced turnovers. Interaction effects revealed that guards benefited most from playing at home in assists, while centers showed the greatest home-court advantage in blocks. Position-outcome interactions showed centers contributed most to winning through 2-point shooting and rebounding, while guards impacted success through playmaking and reduced turnovers. Technical-tactical performance varies substantially by position, match outcome, and location. Coaches should tailor training to leverage positionspecific strengths while developing strategies to overcome positional weaknesses, especially for away games.

## INTRODUCTION

Basketball is a popular and dynamic sport that demands a combination of physical, technical, and tactical skills from its players [1]. Among these elements, technical-tactical performance plays a crucial role in determining a team’s success on the court [2]. Technicaltactical performance metrics are quantitative measures that evaluate a player’s or team’s performance in various aspects of the game, such as shooting, passing, rebounding, and defensive actions [3, 4]. When these metrics demonstrate a significant relationship with success outcomes like winning matches, they can be considered Key Performance Indicators (KPIs) [5]. These KPIs provide valuable insights into a team’s strengths and weaknesses, enabling coaches and analysts to make informed decisions regarding strategies, player selection, and training programs.

Technical-tactical performance in basketball can be assessed through various metrics, including field goal percentage, assists, turnovers, steals, blocks, and rebounds [5]. These KPIs are not only essential for evaluating individual player performance but also for understanding team dynamics and identifying areas for improvement. Effective shooting, efficient ball movement, and strong defensive play are all critical components of a successful basketball team, and KPIs provide a comprehensive view of these elements [6–8].

Women’s basketball exhibits distinct technical-tactical characteristics that necessitate specific investigation despite sharing fundamental rules with men’s basketball [7, 9]. Recent research has identified unique performance profiles in women’s elite competitions, with different patterns in scoring, ball movement, and defensive approaches compared to men’s basketball [10, 11]. Scanlan et al. [12] found that female players demonstrate different technical execution in fundamental skills, which influences the predominant performance indicators in women’s competitions. These sex-specific aspects are essential to consider when analyzing women’s basketball rather than generalizing findings from men’s research [13].

However, technical-tactical performance is not solely determined by individual skills or team tactics; it is also influenced by contextual factors, such as match outcome, match location, and playing positions [14, 15]. Playing positions in basketball represent specialized roles with distinct technical-tactical responsibilities [16, 17]. Zhai et al. [18] analyzed positional differences in elite female basketball and found that guards consistently demonstrate higher rates of assists, steals, and three-point attempts, reflecting their perimeter-oriented playmaking role. Conversely, Fernández-Cortés et al. [3] demonstrated that centers and power forwards exhibit substantially higher rates of two-point field goals, rebounds, and blocks. In women’s basketball specifically, female centers and power forwards show more pronounced specialization in interior play compared to perimeter players, while female guards demonstrate greater emphasis on methodical playmaking [9, 19].

Technical-tactical performances determine match outcomes in basketball, not vice versa [20]. Specific KPIs strongly predict success across women’s basketball competitions, with Leicht et al. [21] finding that effective field goal percentage, defensive rebounds, and assist-to-turnover ratio were the strongest predictors of victory in Olympic women’s basketball. Similarly, Yi et al. [22] demonstrated that successful teams in women’s elite competitions consistently outperform opponents in shooting efficiency and ball security. These performance-outcome relationships appear consistent across different competitive levels in women’s basketball, though the relative importance of specific indicators may vary [7, 9].

Match location represents another critical contextual factor influencing basketball performance [23]. Gómez et al. and García et al. [24, 25] examined Spanish professional leagues and found that home teams consistently demonstrate higher field goal percentages and improved defensive metrics compared to when playing away. These advantages stem from factors including familiarity with the playing environment, supportive crowd influence, reduced travel fatigue, and potential officiating bias [26]. Also, home teams show particularly improved performance in assists and reduced turnovers [8]. Recent research by Sansone et al. [27, 28] has further demonstrated that travel demands and game scheduling interact with location effects, potentially impacting women’s basketball performance more significantly due to typically limited recovery resources [29].

To comprehensively analyze the effects of these contextual factors on technical-tactical performance, linear mixed models can be employed. Unlike traditional statistical analyses like MANOVA or ANOVA, which assume independence of observations, linear mixed models account for the nested structure of the data and can control for the random effects of individual subjects [30]. This is particularly important in longitudinal or repeated measures designs, where observations from the same player are likely to be correlated. By modeling fixed effects (e.g., playing position, match outcome) and random effects (e.g., individual player variability), linear mixed models provide a more accurate estimation of the relationships between variables, reduce the risk of Type I errors, and allow for the generalization of findings beyond the specific sample [31].

Finally, while research has examined technical-tactical performance in basketball, significant gaps exist in our understanding of how contextual factors interact specifically in women’s basketball. Previous studies have typically focused on men’s competitions [6, 17, 32] or analyzed these factors in isolation [15, 29], without accounting for their combined effects or the nested nature of performance data. Few studies have employed appropriate statistical methods like linear mixed modeling that control for individual player variability when examining these relationships over extended periods [30, 31]. This knowledge gap is particularly notable given the potential differences in playing styles and tactical approaches between men’s and women’s basketball [13, 18, 19]. This knowledge gap is particularly notable given the growing recognition that women’s basketball possesses distinct tactical patterns and performance characteristics [13, 19]. The expansion and increased professionalization of women’s basketball have led to evolving playing styles that require contemporary analyses [33]. While recent research has begun to address aspects of performance in women’s basketball [21, 22, 34], comprehensive longitudinal studies examining the interaction between playing position and contextual factors remain scarce [35].

Therefore, the present study aims to investigate the influence of match outcome, match location, and playing positions on the technical-tactical performance of Spanish Female Basketball First Division players during ten seasons (2013–2022). The findings from this research will have direct practical applications: coaches can develop position-specific training programs targeting the technical-tactical elements most critical for success; game strategies can be adjusted based on position-specific advantages in home versus away contexts; and performance analysts can create more contextually-informed evaluation metrics for female players. By employing a mixedmethods approach, this research seeks to contribute to the existing body of knowledge in basketball performance analysis. The main hypotheses of this study were that match outcome, match location and playing positions influence the technical-tactical performance with better results in winning and home condition, as well as in on paint players.

## MATERIALS AND METHODS

### Design

The present study followed a descriptive, observational, relational and associative design, with a retrospective approach analyzing existing competition data [36]. The research protocol was reviewed and approved by the Ethics Committee of the University of Extremadura (reference number: 22/2023) and was conducted in accordance with the Declaration of Helsinki. A linear mixed model was employed to investigate the influence of match outcome (win/ loss), match location (home/away), and playing positions (guard, shooting-guard, small-forward, power-forward and center) on the technical-tactical performance of the Spanish Female Basketball First Division (Liga Femenina Endesa). 1786 regular phase games were registered in ten seasons (2012–2013 to 2021–2022). Data collection will involve different key technical-tactical performance indexes. These performance metrics were dependent variables, while match outcome, match location, and playing positions were the independent variables.

### Sample

The present study focused on the Spanish Women’s Basketball League, known as Liga Endesa Femenina. The target population comprised all players participating in this league over ten consecutive seasons (from 2012–2013 to 2021–2022). A highly representative sample was selected, encompassing 1786 games and 897 basketball players, which generated a total of 33243 individual cases for analysis. These matches included only the regular league phase games, but not considered playoffs, Super Cup, and Queen’s Cup due to the different performance profiles in these contexts [27, 28]. The composition of teams and their respective players experienced variations throughout the study period, reflecting the natural dynamics of the competition. Data were collected on all interventions of each player across all participating teams during the ten seasons.

### Variables

Three independent variables were used for analysis that comprises: (a) playing position, defined as the role of the player during the game, classified into five positions (G: guard; PG: point-guard; SF: smallforward; PF: power-forward; and C: center); (b) match outcome, defined as the final score of the game, to analyze how players perform under different game results (winning vs losing) due to technicaltactical performance determine match outcome [20]; and (c) match location, defined as the venue where the game was played, categorized as either home or away [1, 14]. Conversely, key technicaltactical performance indices (KPIs) were analyzed as dependent variables. Table 1 shows the 16 KPIs evaluated in the present study, that were selected based on their established discriminatory power in women’s basketball performance [21, 22].

**TABLE 1:** Description of KPIs.

Variable	Acronym	Description
Total points	PTS	Total points achieved during the game.
Successful 2-point shots	2PTS	Points scored through successful 2-point field goal attempts.
2-point attempts	2PT	Refers to 2-point field goal attempts that do not result in a basket.
Successful 3-point shots	3PTS	Points scored through successful 3-point field goal attempts.
3-point attempts	3PT	Refers to 3-point field goal attempts that do not result in a basket.
Successful free throws	FTS	Points scored through successful free throw attempts.
Free throws attempts	FT	Refers to free throw attempts that do not result in a point.
Offensive rebounds	OREB	The action of retrieving the ball when a team shoots at the basket and misses; in this case, the attacking team recovers the ball.
Defensive rebounds	DREB	The action of retrieving the ball when a team shoots at the basket and misses; in this case, the defending team secures the rebound.
Assists	AST	Passes from one player to another that directly lead to a successful basket.
Steals	STL	When a player regains possession of the ball from the opposing team.
Turnovers	TO	The number of times a player loses possession of the ball while attempting any action.
Blocks made	BLKm	The action of preventing an opponent from shooting; in this case, the player is the one performing the block.
Blocks against	BLKa	The action of being prevented from shooting; in this case, the player is the one being blocked.
Fouls committed	FLc	Fouls made by a player against an opponent during the game.
Fouls drawn	FLd	Fouls received by a player, committed by the opposing team.

### Procedures

KPIs were initially identified from the FIBA (International Basketball Federation) website (https://www.fiba.basketball) based on their relevance to the research objectives. After determining the seasons to be investigated and the essential indicators for the study, data collection was realized from the official website of the Liga Endesa Femenina (https://www.lfendesa.es/resultados.aspx) and the selected KPIs were gathered and downloaded. Data from the Liga Endesa Femenina official website are derived directly from validated official game scoresheets. This information was then transferred to Microsoft Excel spreadsheets for clear and efficient organization in a large database. To ensure reliability, 90 randomly selected games (5% of total) were independently analyzed by two researchers, yielding Cohen’s kappa values > 0.95 for all variables. Then, the database was imported in .xlsx format to the statistical software for further analysis.

### Statistical analysis

Data was presented as mean and standard deviation. All KPIs were standardized by dividing each player’s raw values by their individual playing time in minutes, allowing for fair comparisons between players regardless of their court time. Differences in KPIs based on playing positions, match location and match outcome were analyzed by linear mixed model [30]. In this model, the aleatory factor of individual response of the female basketball players was controlled including player ID as a random factor. It shows improvements in *R*^2^ conditional (individual players) in comparison with *R*^2^ marginal in all KPIs (*p* < 0.001). In addition, the ICC values were above 0.10 in all KPIs so mixed model should be used. Bonferroni correction was employed for post-hoc comparisons. For effect size of differences, omega partial squared (ω_p_^2^) was used to quantify the magnitude of differences in the main effects and interactions from the linear mixed model (playing position, match outcome, match location, and their interactions), while Cohen’s d was implemented to assess the magnitude of differences in the post-hoc pairwise comparisons between specific groups. Omega partial squared (ω_p_^2^) was interpreted as: < 0.01 trivial, 0.01–0.06 small, 0.06–0.14 medium, > 0.14 large; while Cohen’s was interpreted as: < 0.20 trivial, 0.20–0.60 small, 0.60–1.20 moderate, high (1.2–2.0), and very high (> 2.00) [37]. All statistical analysis was performed by Jamovi (version 2.5, The Jamovi Project, Sidney, Australia) and figures by GraphPad Prism (version 9.5.1, GraphPad Software, Boston, United States).

## RESULTS

### KPIs profile of First Division Female basketball in Spain

Female basketball players during the 2013–2022 seasons participated an average of 21.59 ± 9.97 minutes per match and performed 7.11 ± 5.99 points (0.30 ± 0.22 per minute), 2.10 ± 2.16 successful 2-points throws in 4.62 ± 3.81 2-points attempts (per minute, 2PTS = 0.09 ± 0.09, 2PT = 0.20 ± 0.14), 0.60 ± 0.98 successful 3-points throws in 1.93 ± 2.16 3-points attempts (per minute, 3PTS = 0.03 ± 0.05, 3PT = 0.09 ± 0.10), 1.11 ± 1.63 successful free throws in 1.54 ± 2.09 free throws attempts (per minute, FTS = 0.05 ± 0.08, FT = 0.07 ± 0.10), 0.96 ± 1.31 offensive and 2.56 ± 2.35 defensive rebounds (per minute, OREB = 0.04 ± 0.07, DREB = 0.11 ± 0.10), 1.35 ± 1.62 assists (per minute, AST = 0.05 ± 0.08), 0.85 ± 1.09 steals (per minute, STL = 0.04 ± 0.06), 1.54 ± 1.50 turnovers (per minute, TO = 0.07 ± 0.08), 0.19 ± 0.51 blocks made and 0.18 ± 0.46 blocks against (per minute, BLKm = 0.01 ± 0.03, BLKd = 0.01 ± 0.03), 1.79 ± 1.25 fouls committed and 1.78 ± 1.78 fouls received (per minute, FLc = 0.09 ± 0.10, FLd = 0.08 ± 0.08). The shooting percentage was 43.03 ± 29.07% for 2-points throws, 28.54 ± 31.79% for 3-points throws, and 71.34 ± 30.69% for free throws.

### Playing positions

**FIG. 1:**
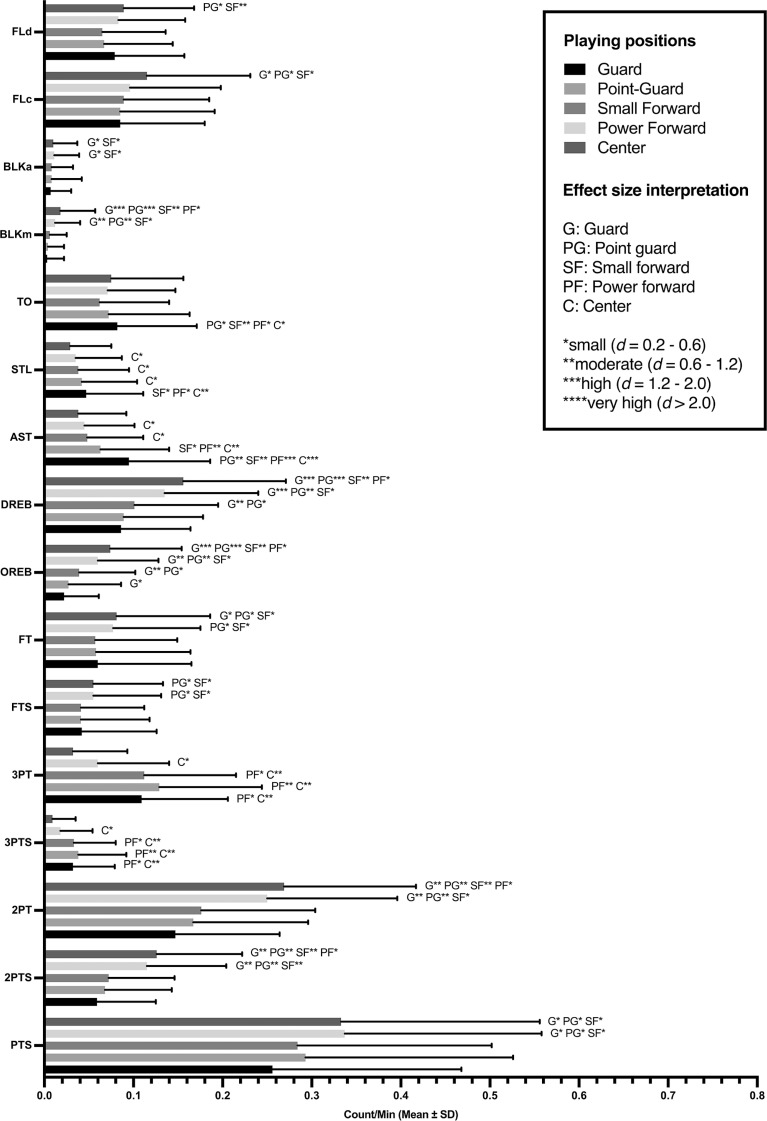
Descriptive and statistical differences in KPIs between playing positions. Note: PTS: Total points, 2PTS: 2-points successful, 2PT: 2-point attempts, 3PTS: 3-points successful, 3PT: 3-point attempts, FTS: free-throw successful, FT: free throws attempts, OREB: offensive rebounds, DREB: defensive rebounds, AST: assists, STL: steals, TO: turnovers, BLKm: blocks made, BLKa: blocks against, FLc: fouls committed, FLd: fouls drawn.

All KPIs presented statistical differences based on playing positions with large effect size in DREB, OREB, AST, BLKm, 2PTS and 2PT (*F* = 31.50-to-73.60; ω_p_^2^ = 0.15-to-0.29; *p* < 0.01), medium effect size in 3PTS, 3PT and STL (*F* = 12.40-to-26.10; ω_p_^2^ = 0.06-to-0.12; *p* < 0.01), and small effect size in FLd, PTS, FT, TO, FLc, FTS and BLKa (*F* = 5.43-to-11.10; ω_p_^2^ = 0.02-to-0.05; *p* < 0.01). In post-hoc comparisons, centers obtained higher values in 2PTS, 2PT, OREB, DREB, BLKm, and FLc; guards in AST and TO; guards and point-guards in 3PTS, 3PT, and STL; and power forwards and centers in PTS, FTS, FT, BLKa, and FLd. Figure 1 represents the descriptive analysis of KPIs and the effect size of differences between playing positions.

### Game outcome by playing positions

The game outcome reported statistical differences in all KPIs with large effect size in PTS, AST, 2PTS, DREB and 3PTS (*F* = 218.60-to-617.15; ω_p_^2^ = 0.20-to-0.41; *p* < 0.01), medium effect size in STL, FTS and TO (*F* = 75.26-to-98.56; ω_p_^2^ = 0.08-to-0.10; *p* < 0.01), and small effect size in FT, OREB, BLKm, BLKa, 3PT and 2PT (*F* = 8.73-to-54.48; ω_p_^2^ = 0.01-to-0.06; *p* < 0.01). No significant differences were found in FLc and FLd (*F* = 0.51-to-0.80; ω_p_^2^ < 0.01; *p* > 0.05). The combination of game outcome with playing positions showed statistical differences with small effect size in 2PTS, 2PT, 3PTS, 3PT, OREB, DREB, AST, and BLKm (*F* = 4.85-to-14.10; ω_p_^2^ = 0.02-to-0.06; *p* < 0.01), and no significant differences in PTS, FTS, FT, STL, TO, BLKa, FLc, and FLd (*F* = 0.70-to-2.31; ω_p_^2^ < 0.01; *p* > 0.05).

**FIG. 2:**
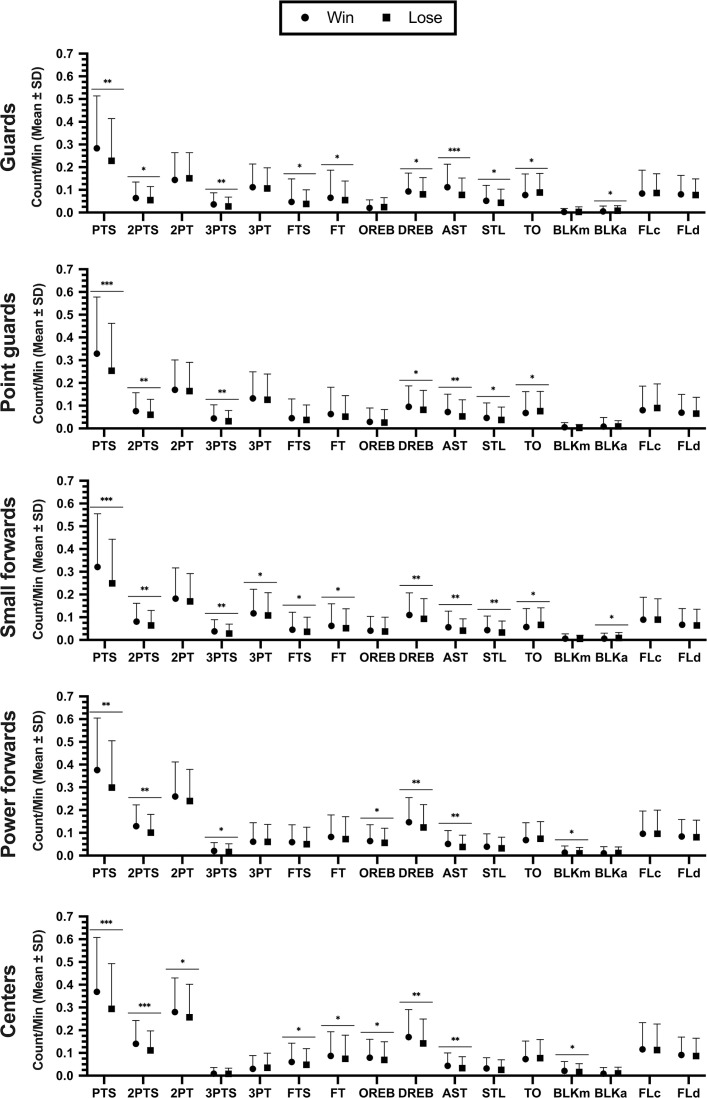
Dots plot to represent the effect of match outcome in KPIs per player position. Effect size: *small (*d* = 0.20-to-0.60), **moderate (*d* = 0.60-to-1.20), ***high (*d* = 1.20-to-2.00). Note: PTS: Total points, 2PTS: 2-points successful, 2PT: 2-point attempts, 3PTS: 3-points successful, 3PT: 3-point attempts, FTS: free-throw successful, FT: free throws attempts, OREB: offensive rebounds, DREB: defensive rebounds, AST: assists, STL: steals, TO: turnovers, BLKm: blocks made, BLKa: blocks against, FLc: fouls committed, FLd: fouls drawn.

Figure 2 shows the post-hoc comparisons by playing positions. Statistical differences were reported in order of importance: (a) guards with high effect size in AST (*d* = 1.46), with moderate effect size in PTS, 3PTS and STL (*d* = 0.62-to-1.01), and with low effect size in TO, DREB, FTS, FT, 2PTS and BLKa (*d* = 0.37-to-0.58); (b) point guards with high effect size in PTS (*d* = 1.24), with moderate effect size in 3PTS, AST and 2PTS (*d* = 0.65-to-1.11), and low effect size in STL, DREB and TO (*d* = 0.45-to-0.54); (c) small forwards with high effect size in PTS (*d* = 1.26), with moderate effect size in 3PTS, AST, DREB, 2PTS and STL (*d* = 0.61-to-0.96), and with low effect size in 3PT, TO, FTS, FT and BLKa (*d* = 0.35-to-0.50); (d) power forward with moderate effect size in PTS, 2PTS, DREB and AST (*d* = 0.76-to-1.17), and with low effect size in OREB, 3PTS and BLKm (*d* = 0.38-to-0.42); (e) centers with high effect size in 2PTS and PTS (*d* = 1.25-to-1.28), with moderate effect size in DREB, BLKm and AST (*d* = 0.63-to-1.11), and with low effect size in FTS, OREB, FT and 2PT (*d* = 0.39-to-0.59).

No differences were found in: (a) guards in 2PT, OREB and BLKm; (b) point guards in 2PT, FTS, FT, OREB, BLKm and BLKa; (c) small forwards in 2PT, OREB and BLKm; (d) power forwards in 2PT, FTS, FT, STL, TO and BLKa; and (e) centers in 3PTS, STL, TO, and BLKa. In all playing positions, no differences were found in 3PT, FLc and FLd.

### Game location by playing positions

The game location reported statistical differences with large effect size in AST (*F* = 243.00; ω_p_^2^ = 0.21; *p* < 0.01), medium effect size in BLKm and TO (*F* = 78.34; ω_p_^2^ = 0.08; *p* < 0.01), and small effect size in STL, PTS, BLKa, TO, OREB, 2PTS, DREB, 3PT and 3PTS (*F* = 14.93-to-48.23; ω_p_^2^ = 0.02-to-0.05; *p* < 0.01). No differences were found in 2PT, FLc and FLd (*F <* 4.28; ω_p_^2^ < 0.01; *p* > 0.05). In the other hand, the combination of game location with playing positions shown statistical differences with medium effect size in AST (*F* = 20.80; ω_p_^2^ = 0.08; *p* < 0.01), and small effect size in BLKm, OREB and 3PT (*F* = 2.53-to-7.06; ω_p_^2^ = 0.01-to-0.03; *p* < 0.04). No differences were found in PTS, 2PTS, 2PT, 3PTS, FTS, FT, DREB, STL, TO, BLKa, FLc and FLd. (*F <* 1.86; ω_p_^2^ < 0.01; *p* > 0.11).

**FIG. 3:**
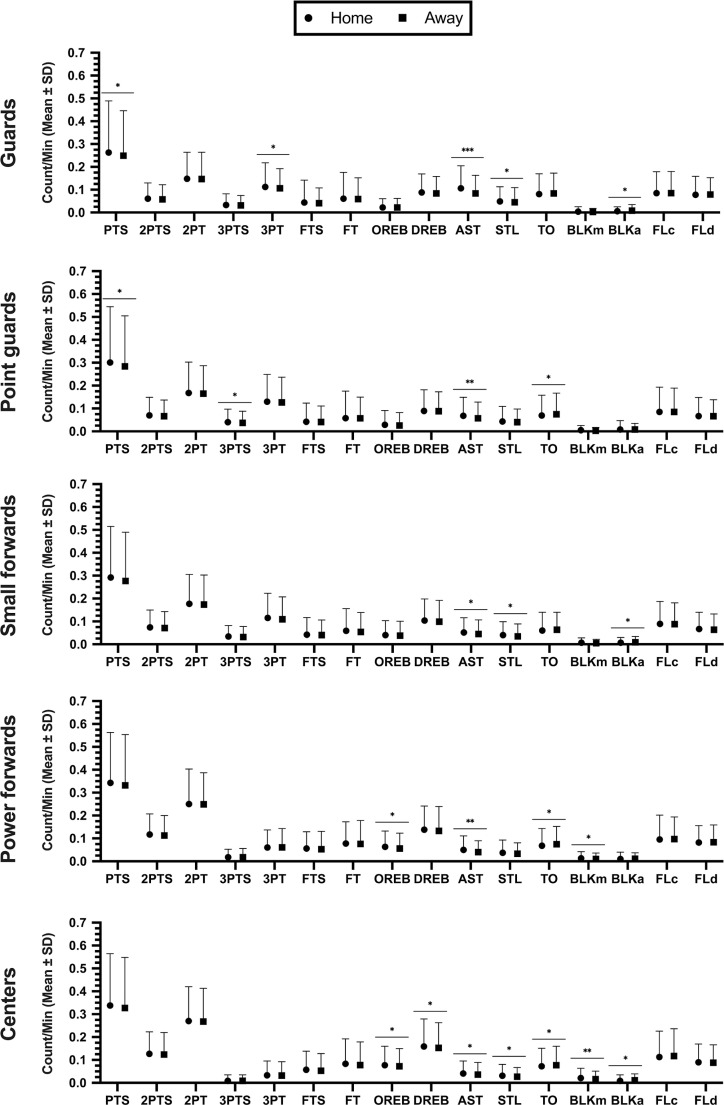
Dots plot to represent the effect of match location in KPIs per player position. Effect size: *small (*d* = 0.20-to-0.60), **moderate (*d* = 0.60-to-1.20), ***high (*d* = 1.20-to-2.00). Note: PTS: Total points, 2PTS: 2-points successful, 2PT: 2-point attempts, 3PTS: 3-points successful, 3PT: 3-point attempts, FTS: free-throw successful, FT: free throws attempts, OREB: offensive rebounds, DREB: defensive rebounds, AST: assists, STL: steals, TO: turnovers, BLKm: blocks made, BLKa: blocks against, FLc: fouls committed, FLd: fouls drawn.

Figure 3 shows the post-hoc comparisons by playing positions. Statistical differences were reported in order of importance: (a) guards with high effect size in AST (*d* = 1.50), and with small effect size in 3PT, BLKa, PTS and STL (*d* = 0.32-to-0.36); (b) point guards with moderate effect size in AST (*d* = 0.73), and with small effect size in PTS, 3PTS and TO (*d* = 0.36-to-0.48); (c) small forwards with small effect size in AST, STL and BLKa (*d* = 0.37–0.45); (d) power forwards with moderate effect size in AST (*d* = 0.68), and with small effect size in OREB, BLKm and TO (*d* = 0.38-to-0.51); (e) and centers with moderate effect size in BLKm (*d* = 0.84), and with small effect size in OREB, AST, STL, DREB and BLKa (*d* = 0.33-to-0.39).

No significant differences were found in: (a) guards in 3PTS, DREB, OREB, TO and BLKm; (b) point guards in 3PT, OREB, DREB, STL, BLKm and BLKa; (c) small forwards in PTS, 3PTS, 3PT, OREB, DREB, TO and BLKm; (d) power forwards in PTS, 3PTS, 3PT, DREB, STL y BLKa; and (e) centers in PTS, 3PTS, 3PT and TO. In all playing positions, no differences were found in 2PTS, 2PT, FTS, FT, FLc and FLd.

## DISCUSSION

Analysis of 1786 games and 33243 individual cases across ten seasons of Spanish elite women’s basketball (2013–2022) revealed distinctive technical-tactical patterns that vary significantly by context. Our findings revealed three key novel contributions: 1) positionspecific performance patterns where centers and power-forwards excel in paint-related actions while guards dominate perimeter play; 2) distinct technical-tactical profiles characterizing winning teams, particularly in points, assists, and reduced turnovers; and 3) significant home-court advantages, especially for guards in playmaking and centers in defensive actions. Notably, our findings revealed significant interaction effects, with centers contributing most significantly to winning outcomes through rebounding and interior scoring, while guards’ impact on success was most pronounced through playmaking and reduced turnovers. These results extend beyond previous research by identifying not just isolated positional or contextual effects, but their specific interactive patterns in women’s basketball using rigorous mixed modeling techniques that account for individual player variability.

### Playing positions

Our findings revealed distinct technical-tactical profiles for each playing position, with the most differences occurring in paint-related actions for centers and perimeter play for guards. These findings are consistent with previous research in men’s basketball [1, 17, 20, 35] and recent studies in women’s basketball [3, 18], highlighting the specialized roles within a basketball team. Centers and power forwards dominated in 2-point shooting, rebounding, and shot-blocking, while guards excelled in 3-point shooting, assists, and steals. These position-specific profiles reflect the tactical responsibilities assigned to each position and the physical attributes typically associated with players in these roles [34].

The pronounced differences in paint-related actions underscore the importance of “big” players (centers and power forwards) in controlling the paint area. Their height and physical presence allow them to score from close range, secure rebounds, and contest opponents’ shots effectively [16]. This spatial dominance creates tactical advantages through second-chance opportunities and defensive deterrence, fundamentally altering offensive and defensive schemes around the basket. These findings align with Yi et al. [22], who reported that centers in the Women’s Chinese Basketball Association (WCBA) had significantly higher offensive and defensive rebound rates compared to other positions.

The perimeter specialization of guards reflects their role in perimeter play and orchestrating the offense [6]. This tactical positioning enables them to stretch defensive coverage, create spacing for interior players, and initiate offensive sequences through superior court vision and ball-handling skills. This is consistent with Zhai et al. [18], who found that elite female guards in China had higher usage rates and assist rates compared to other positions. Interestingly, while our results show clear positional differences in 3-point shooting, some studies in men’s basketball have reported a trend towards positional convergence in this aspect [38], suggesting that the specialization in 3-point shooting might be more pronounced in women’s basketball.

Our study also revealed that guards had the highest turnover rates, a finding that, while perhaps counterintuitive, can be explained by their high ball possession time and the defensive pressure they face as primary ball handlers [10]. This risk-reward dynamic is inherent to their tactical role, as increased ball-handling responsibility inevitably exposes them to more defensive pressure and decision-making situations under time constraints. Madarame [39] found similar results in the NBA where turnovers were positively correlated with assists, implying that players who handle the ball more (typically guards) are prone to more turnovers. However, the risk-reward balance of this high-involvement style of play is evident in our findings, as guards also lead in assists and steals. The tactical implication is that successful teams must develop guards with superior decision-making under pressure while implementing offensive systems that provide adequate support options to reduce turnover risks. It is worth noting that our analysis did not account for the timing of turnovers within the game, the score at the time of the turnover, or factors such as physical fatigue. Sampaio et al. [20] suggest, these contextual factors can significantly influence player performance and decision-making, potentially affecting turnover rates at critical moments of the game.

The differences in KPIs across playing positions underscore the need for position-specific training and performance analysis. Ferioli et al. [40] demonstrated in their study on the physical demands of different playing positions in basketball, tailored conditioning programs are crucial for optimizing performance. Similarly, our findings suggest that coaches should design specific drills to improve ball handling and decision-making under pressure for guards, while developing post-play and rebounding skills for forwards and centers. Moreover, the positional differences we observed have tactical implications. The complementary nature of interior and perimeter specialization creates tactical interdependencies that successful teams must optimize. The strong inside presence of centers and power forwards, coupled with the perimeter threat posed by guards, creates a spatial distribution that is crucial for offensive spacing [41]. Coaches can leverage these position-specific strengths to create tactical mismatches, implement pick-and-roll combinations that exploit size advantages, and design floor spacing that maximizes each player’s spatial effectiveness.

### Match outcome effects

The performance profiles of winning teams were characterized by significantly superior execution across all technical-tactical indicators, demonstrating tactical superiority in both offensive efficiency and ball movement. This is consistent with findings from men’s basketball [6, 7] and women’s basketball [7, 21]. The position-specific contributions to winning outcomes provided insights into position-specific contributions to winning. However, it is important to note that while our analysis focuses on individual positions, basketball is inherently a team sport where success depends on the synergy between different positions. Courel-Ibáñez et al. [33] mentioned that the interactions between players and the collective strategies employed may be more influential in determining match outcomes than the performance of individual positions in isolation. This suggests that winning teams successfully integrate individual positional strengths into cohesive tactical systems. While performance indicators are attributed to individual players, the advantages that lead to these outcomes often result from coordinated team efforts.

Winning teams demonstrated tactical superiority through enhanced interior scoring by centers and superior playmaking by guards. These findings align with Gómez et al. [7] and Leicht et al. [21], who highlighted the importance of effective post play and efficient close-range shooting in women’s basketball success. The tactical implication is that successful teams establish interior dominance through their centers while maintaining perimeter control through guard play, creating a balanced offensive approach that stresses multiple areas of the defense. The enhanced performance of guards in winning teams supports Mavridis et al. [42] emphasis on guard play’s importance in basketball victories.

Interestingly, we found reduced differences in 3-point shooting between winning and losing teams across all positions, contrasting with men’s basketball studies where 3-point shooting often discriminates winning teams [6, 17]. This suggests that tactical success in women’s basketball may rely more heavily on interior efficiency and ball movement rather than perimeter shooting volume, indicating different strategic priorities compared to men’s basketball, possibly due to differences in physical attributes, tactical approaches, or relative skill levels in long-range shooting between men’s and women’s basketball [32].

However, it’s important to note that women’s basketball is played under rules primarily designed for men, with only minor modifications such as ball size, which may influence playing strategies and efficacy. These rule constraints may partially explain the tactical emphasis on interior play and methodical offensive approaches observed in successful women’s teams. These findings collectively suggest that successful teams optimize each playing position’s strengths while minimizing weaknesses. From a tactical perspective, coaches should focus on maximizing centers’ and power forwards’ offensive efficiency near the basket while enhancing guards’ playmaking abilities and ball security. This position-specific approach to game planning is supported by Dehesa et al. [43], who found significant variations in performance profiles by playing position and game context in elite basketball.

### Match location effects

Spanish elite-level female basketball supports the well-documented home advantage phenomenon in sports [23]. The pronounced home advantage in ball movement and defensive aggression suggest that home teams play more cohesively on offense and more aggressively on defense. This tactical advantage manifests across multiple performance dimensions, suggesting that familiar environments enable more confident and coordinated team play. These findings are consistent with studies in both men’s and women’s basketball across different countries and competitive levels [24, 25, 44].

The differential impact of home advantage across positions reveals tactical implications for game planning. Guards demonstrate enhanced playmaking confidence at home, likely benefiting from familiar sight lines and crowd support for decision-making. Centers show increased defensive aggression, suggesting that home environments enable more assertive interior defense. While positional variation in home advantage has been less explored, this finding can be interpreted through the lens of home advantage mechanisms such as familiarity with the court, supportive crowd, and reduced travel fatigue [45]. The tactical advantage for interior players at home appears to stem from increased comfort with spatial positioning and rim protection, while perimeter players benefit from enhanced court vision and passing accuracy. These findings partially align with Gómez et al. [7] identification of defensive rebounds as key predictors of home wins in Spanish professional basketball.

Despite the significance of home advantage, its magnitude varies across sports. Jamieson [46] realized a meta-analysis that found robust home advantage in basketball, which is not as pronounced in some other team sports. Our study contributes by quantifying effect sizes for specific technical-tactical actions and playing positions in women’s basketball. These findings suggest that coaches should emphasize aggressive defense and fluid offense in home games, particularly leveraging guards’ improved playmaking. For away games, strategies should focus on minimizing the hostile environment’s impact, such as using timeouts to disrupt the home team’s rhythm [7], simplifying offensive sets, prioritizing defensive rebounding, and implementing mental preparation strategies [32].

### Limitations and future research directions

While this study provides valuable insights into the technical-tactical performance of Spanish female basketball players, several limitations should be considered. The analysis is confined to one national league, which may limit the generalizability of findings to other countries or competitive levels. The study focused solely on game-related statistics without accounting for physical, physiological, or psychological factors that could influence performance. Additionally, the quality of opposition and specific team strategies were not considered, which might affect the interpretation of some results. The retrospective nature of the data also prevents the establishment of causal relationships.

Furthermore, the study period (2013–2022) included COVID-19 pandemic seasons, although it should be noted that when COVID-19 disrupted competition, the league finished without playoffs, and our analysis only included regular phase games across all seasons for consistency. Additional contextual factors such as travel demands, game scheduling (back-to-back games, rest days), opponent quality, and potential differences between regular season and playoff performances were not accounted for in the current analysis. It is also important to note that these findings may not be generalizable to youth competitions or men’s basketball, where different technical-tactical patterns might emerge due to physical development stages or sex-specific playing styles.

Future research could address these limitations by extending the analysis to other leagues, integrating multidimensional performance indicators, and employing longitudinal designs to track individual and team development over time. Incorporating spatiotemporal data could provide insights into the tactical nuances of different playing positions, while mixed-methods approaches, including qualitative research with coaches and players, could enhance our understanding of the decision-making processes underlying the observed statistical trends. Such advancements would contribute to a more comprehensive understanding of performance in women’s basketball and bridge the gap between research and practical application.

## CONCLUSIONS

Playing positions significantly influence all KPIs in Spanish elite women’s basketball, especially in 2-point shots, offensive and defensive rebounds and blocks against. Winning teams demonstrated superior performance across most indicators, particularly in points, assists and turnovers, reflecting the technical-tactical elements that distinguish successful outcomes. Home advantage was found, especially in assists, blocks and turnovers, indicating more cohesive offensive play and aggressive defense at home condition. By playing positions, guards benefit the most from playing at home, showing a substantial increase in assists, while centers and power forwards improve in offensive rebounding and shot-blocking. Finally, 3-point shooting does not strongly discriminate between winning and losing teams across positions.

From these results, different practical applications could be given for team staff: *(1) Position-specific training:* coaches should design tailored training programs that focus on enhancing the KPIs for each playing position (e.g., emphasize close-range shooting and rebounding for centers, ball-handling and decision-making under pressure for guards); *(2) Offensive strategies:* Given the importance of 2-point shooting and assists in winning, coaches should develop offensive plays that prioritize high-percentage shots near the basket and facilitate ball movement to create open looks, especially for centers and power forwards; *(3) Defensive focus:* Since winning teams excel in blocks and defensive rebounds, coaches should implement defensive schemes that firstly pressure after turnovers and then protect the paints and secure defensive rebounds. This could involve practicing boxing-out techniques and defensive rotations; *(4) Home game preparation:* To maximize home advantage, coaches should design training sessions that simulate home-game conditions (e.g., with crowd noise), and special attention should be given to enhancing guards’ playmaking abilities and centers’ offensive rebounding during home games; *(5) Away game strategies:* Coaches should prepare simplified offensive sets to reduce turnovers and focus on defensive rebounding to counter the home team’s improved offensive rebounding. Mental preparation techniques to cope with away-game stress should also be incorporated; *(6) Timeout management:* Coaches should strategically use timeouts in away games to disrupt the home team’s rhythm, especially when the opposition’s guards are effectively distributing the ball; *(7) Performance analysis:* Regular monitoring of these key performance indicators can help coaches identify areas of improvement for each player and the team as a whole, as well as can inform in-season training adjustments and player rotation decisions; and *(8) Recruitment:* Team managers and coaches should prioritize to sign centers with strong 2-point shooting and rebounding abilities, and guards with high assist-to-turnover ratios, as these skills are crucial for team success.

**Figure:**